# Commentary on: A Call to Action: Facing the Shadow Pandemic of Complicated Forms of Grief

**DOI:** 10.1177/00302228211016227

**Published:** 2021-05-21

**Authors:** Maarten C. Eisma, Paul A. Boelen

**Affiliations:** 1 Department of Clinical Psychology and Experimental Psychopathology, University of Groningen, Groningen, the Netherlands; 2 Department of Clinical Psychology, Utrecht University, Utrecht, the Netherlands; 3 ARQ Psychotrauma Expert Center, Diemen, the Netherlands

**Keywords:** COVID-19, coronavirus, grief, bereavement, prolonged grief disorder, review

## Abstract

In this contribution, we respond to a letter in Omega: Journal of Death and Dying by Doka. Signatories of this letter to the President of the United States convey concerns that deaths during the COVID-19 pandemic will lead to a higher prevalence of severe and persistent grief, i.e., prolonged grief disorder. We support their call to action to direct government funding to helping those who develop this condition during the COVID-19 pandemic. However, we think that concerns about prolonged grief disorder during the pandemic can be more convincingly conveyed by firmly embedding such concerns within scientific literature. Therefore, we highlight prior scientifically informed opinion pieces from various international researchers who voiced similar concerns in the early months of the pandemic. Additionally, we provide an overview of pioneering empirical research elucidating whether prolonged grief disorder and related mental health problems will become more prevalent during the pandemic.

With interest we have read Doka’s (2021) letter to the President of the United States titled ‘A Call to Action: Facing the Shadow Pandemic of Complicated Forms of Grief’ recently published in Omega: Journal of Death and Dying. We share the concerns voiced by signatories of this letter that the characteristics and circumstances of deaths due to COVID-19 will likely precipitate a heightened prevalence of severe, persistent, and disabling grief, also termed prolonged grief disorder (Eisma et al., 2020). We appreciate the effort of prominent American grief researchers, health care professionals, and policy makers to bring such concerns to the attention of the highest office in the United States. We share their hope that this will increase opportunities to obtain funding to enhance existing therapeutic and hospice services, train healthcare professionals, educate the general public, and conduct clinically-oriented research in order to, somehow, improve the lives of people bereaved during this pandemic.

However, we think that the call for more attention for (disordered) grief during and after the pandemic could be delivered with even greater force by embedding it within scientific literature. Despite citing two scientific sources, the contents of Doka’s (2021) letter appear to primarily reflect ideas and assumptions from the signatories. However, to demonstrate the validity of concerns about rising prevalence of prolonged grief disorder during the pandemic, it is valuable to consider prior scientific writings in this area. Specifically, as this is a global health crisis, it is relevant to acknowledge work of many other international scholars, who cogently voiced similar concerns in the early months of the pandemic. In addition, argumentation for the notion that the prevalence of prolonged grief disorder will rise due to the pandemic may be strengthened by considering recent pioneering research supporting this claim. In what follows, we will expand on these two topics.

We will turn first to the historical development of the viewpoint that COVID-19 deaths may lead to higher prevalence rates of prolonged grief disorder. Notably, Doka’s (2021) letter supersedes many earlier scientifically informed opinion pieces on the topic of grief during the COVID-19 pandemic. Almost a year ago, as the pandemic started to influence the lives of people worldwide, researchers around the world set out to identify how bereavement and grief may be different for people who lose loved ones due to COVID-19.

In April 2020, Wallace et al. (2020) and Eisma et al. (2020) were among the first to share their scientifically informed opinions about the consequences of the pandemic on the grief process. Together, they highlighted most of the potential risk factors for severe and persistent grief reactions experienced by people bereaved due to COVID-19 inventoried at the bottom of the letter by Doka (2021). They hypothesized that unexpected deaths, preceded by intensive care admission, the experience of a variety of secondary stressors (incl. multiple losses or other concurrent losses), a lack of opportunities to say goodbye and shape death rituals, and the reduced accessibility of social support and social isolation, may make grieving more difficult. Each group of researchers also shared unique viewpoints. For example, Wallace et al. (2020) mentioned the possibility of experiencing disenfranchised grief as COVID-19 losses are often not be appropriately publicly mourned or acknowledged. Eisma et al. (2020) highlighted the possibility that people experiencing deaths due to other causes than COVID-19 during the pandemic may be exposed to some of the same risk factors of poor bereavement outcome as those experiencing deaths due to COVID-19.

Other opinion pieces in the first months of the pandemic from researchers worldwide conveyed similar concerns, adding complementary perspectives or more in-depth considerations on particular topics (e.g., Breen, 2020; Carr et al., 2020; Gesi et al., 2020; Goveas & Shear, 2020; Killikelly et al., 2021; Kokou-Kpolou et al., 2020; Simon et al., 2020; Sun et al., 2020; for a review, see Stroebe & Schut, 2020). Acknowledging such perspectives is critical to illustrate how widely supported concerns are regarding the mental health problems of people who become bereaved during the pandemic. Additionally, since these perspectives are scientifically informed, they lay important foundations for most of Doka’s (2021) statements on putative risk factors for prolonged grief disorder.

Encouragingly, researchers have already started conducting the empirical research that is so urgently needed to clarify risks of prolonged grief disorder and related mental health problems among people who become bereaved during the pandemic. We will discuss these studies next, focusing on quantitative research, as this is arguably most relevant to this discussion. Eisma et al. (2021) provided the first empirical data on COVID-19 bereavement and the odds of developing prolonged grief disorder. They showed within a large-scale survey study that acute grief levels are higher among Dutch adults who experienced COVID-19 bereavement, compared to those who experience deaths due to natural causes (e.g., illness), yet comparable to those who experience deaths due to unnatural causes (i.e., accidents, murder, suicide). Since acute grief is predictive of later prolonged grief symptoms (Boelen & Lenferink, 2020) this provides a clear signal that prolonged grief disorder will become more prevalent in people bereaved due to COVID-19. A second study on the same dataset further demonstrated that people who recently experienced a non-COVID-19 death during the pandemic report higher acute grief levels than those who recently experienced bereavement before the pandemic (Eisma & Tamminga, 2020). Therefore, coping with grief during the pandemic may also be more difficult for people bereaved due to other causes than COVID-19.

Another recent study found that acute grief and posttraumatic stress, depression, and anxiety symptoms were elevated among American adults bereaved due to COVID-19 (Breen et al., 2021e; for similar findings on acute grief in an adolescent sample see: Murata et al., 2021). A survey study of Joaquim et al. (2021) in a large sample of Brazilian adults is also of interest here. They demonstrated that people who had experienced COVID-19 related losses experienced more general distress than those who had not experienced such losses. Particularly noteworthy is recent research by Tang and Xiang (2021); based on a survey of Chinese COVID-19 bereaved adults, including a subset who lost someone more than six months ago, they showed that 38% and 29% met criteria for prolonged grief disorder as defined in ICD-11 and proposed by Prigerson et al. (2009), respectively. In further analyses of this same dataset, Tang et al. (2021) reported that adults bereaved due to COVID-19 reported elevated symptom levels posttraumatic stress, anxiety, and depression, alongside prolonged grief disorder.

Together, this seminal work sheds light on the detrimental mental health impact of bereavement during the pandemic as well as risk factors determining poor bereavement outcomes. For example, Eisma et al. (2021) and Eisma and Tamminga (2020) demonstrated that satisfaction with social support did not differ between people experiencing deaths caused by COVID-19 versus other types of deaths nor between people recently bereaved due to other causes than COVID-19 before versus during the pandemic . This appears at odds with claims that social support is a critical explanatory factor of differences in grief reactions between these groups. However, Eisma et al. (2021) did show that the unexpected nature of COVID-19 deaths explained differences in acute grief levels between people who experienced COVID-19 deaths versus natural deaths. Tang et al.’s (2021) multivariate examinations of correlates of mental health in a COVID-19 bereaved sample are also informative, showing that loss of first-degree relatives, feeling traumatized by the loss, and having had a close and/or conflictual relationship with the deceased relate most consistently to poor mental health outcomes. Nevertheless, many putative risk factors of prolonged grief disorder within this population (including those listed by Doka, 2021) have yet to be put to a stringent empirical test.

While these pioneering studies inevitably have certain limitations, such as their predominant reliance on voluntary response sampling and use of cross-sectional online survey data, they show a remarkably consistent pattern of findings regarding the mental health of COVID-19 bereaved adults. Globally, deaths due to COVID-19 are associated with higher levels of acute and prolonged grief and related affective and stress-related symptomatology. Moreover, some factors (e.g., unexpectedness of deaths) appear more likely candidates to be risk factors for prolonged grief disorder within these populations than others (e.g., limitations in social support). Research further tentatively suggests that grief may be more severe and persistent for people who experience non-COVID-19 deaths during the pandemic. Together, this evidentiary base provides an important starting point for anyone involved in research, policy, public education, and professional help aimed at improving the lives of people who lose loved ones during this ongoing health crisis.

In summary, we share Doka's (2021) observation that the COVID-19 pandemic will give rise to a surge of prolonged grief disorder cases. We agree that action is needed to address this problem. In our view, we can add force to this call for action by considering the impressive list of recent scholarly writings in which concerns about the increase in prolonged grief disorder during the pandemic were convincingly articulated and the preliminary empirical evidence for these concerns. Only by painting a complete picture of this research area, can readers, including the President of the United States, be optimally informed about the challenges bereaved people face during this unprecedented global disaster.

## References

[bibr1-00302228211016227] Boelen P. A. Lenferink L. (2020). Symptoms of prolonged grief, posttraumatic stress, and depression in recently bereaved people: Symptom profiles, predictive value, and cognitive behavioural correlates. Social Psychiatry and Psychiatric Epidemiology, 55(6), 765–777. 10.1007/s00127-019-01776-w 31535165 PMC7275015

[bibr2-00302228211016227] Breen L. J. (2020). Grief loss and the COVID-19 pandemic. Australian Journal of General Practice, 49. 10.31128/ajgp-covid-20 32416647

[bibr3-00302228211016227] Breen L. J. Lee S. A. Neimeyer R. A. (2021). Psychological risk factors of functional impairment after COVID-19 deaths. Journal of Pain and Symptom Management, 61(4), e1–e4. 10.1016/j.jpainsymman.2021.01.006 33476753

[bibr4-00302228211016227] Carr D. Boerner K. Moorman S. (2020). Bereavement in the time of coronavirus: Unprecedented challenges demand novel interventions. Journal of Aging & Social Policy, 32(4-5), 425–431. 10.1080/08959420.2020.1764320 32419667

[bibr5-00302228211016227] Doka K. (2021). A call to action: Facing the shadow pandemic of complicated forms of grief. OMEGA-Journal of Death and Dying, 83(1), 164–169. 10.1177/0030222821998464 33736545

[bibr6-00302228211016227] Eisma M. C. Boelen P. A. Lenferink L. I. M. (2020). Prolonged grief disorder following the coronavirus (COVID-19) pandemic. Psychiatry Research, 288, 113031. 10.1016/j.psychres.2020.113031 32360895 PMC7194880

[bibr7-00302228211016227] Eisma M. C. Tamminga A. (2020). Grief before and during the COVID-19 pandemic: Multiple group comparisons. Journal of Pain and Symptom Management, 60(6), e1–e4. 10.1016/j.jpainsymman.2020.10.004 PMC7553097 33065207

[bibr8-00302228211016227] Eisma M. C. Tamminga A. Smid G. E. Boelen P. A. (2021). Acute grief after deaths due to COVID-19, natural causes and unnatural causes: An empirical comparison. Journal of Affective Disorders, 278, 54–56. 10.1016/j.jad.2020.09.049 32950843 PMC7487144

[bibr9-00302228211016227] Gesi C. Carmassi C. Cerveri G. Carpita B. Cremone I. M. Dell’Osso L. (2020). Complicated grief: What to expect after the coronavirus pandemic. Frontiers in Psychiatry, 11, 489. 10.3389/fpsyt.2020.00489 32574243 PMC7264152

[bibr10-00302228211016227] Goveas J. S. Shear M. K. (2020). Grief and the COVID-19 pandemic in older adults. The American Journal of Geriatric Psychiatry, 28(10), 1119–1125. 10.1016/j.jagp.2020.06.021 32709542 PMC7320675

[bibr11-00302228211016227] Joaquim R. M. Pinto A. L. C. B. Guatimosim R. F. de Paula J. J. Souza Costa D. Diaz A. P., da Silva, A.G., Pinheiro, M.I.C., Serpa, A. L. O., Miranda, D. M., & Malloy-Diniz L. F. (2021). Bereavement and psychological distress during COVID-19 pandemics: The impact of death experience on mental health. Current Research in Behavioral Sciences, 2, 100019. 10.1016/j.crbeha.2021.100019

[bibr12-00302228211016227] Killikelly C. Smid G. E. Wagner B. Boelen P. A. (2021). Responding to the new International Classification of Diseases-11 prolonged grief disorder during the COVID-19 pandemic: A new bereavement network and three-tiered model of care. Public Health, 191, 85–90. 10.1016/j.puhe.2020.10.034 33556639

[bibr13-00302228211016227] Kokou-Kpolou C. K. Fernández-Alcántara M. Cénat J. M. (2020). Prolonged grief related to COVID-19 deaths: Do we have to fear a steep rise in traumatic and disenfranchised griefs? Psychological Trauma: Theory, Research, Practice, and Policy, 12(S1), S94–S95. 10.1037/tra0000798 32525367

[bibr14-00302228211016227] Mayland C. R. Harding A. J. E. Preston N. Payne S. (2020). Supporting adults bereaved through COVID-19: A rapid review of the impact of previous pandemics on grief and bereavement. Journal of Pain and Symptom Management, 60(2), e33–e39. 10.1016/j.jpainsymman.2020.05.012 PMC7228694 32416233

[bibr5000-00302228211016227] Murata, S., Rezeppa, T., Thoma, B., Marengo, L., Kranevich, K., Chiyka, E., Hayes, B., Goodfriend, E., Deal, M., Zhong, Y., Brummit, B., Coury, T., Riston, S., Brent, D. A., & Melhem, N. M. (2021). The psychiatric sequalae of the COVID-19 pandemic in adolescents, adults, and healthcare workers. *Depression & Anxiety, 38*, 2, 233–246. https://doi.org/10.1002/da.23120 10.1002/da.23120 PMC7902409 33368805

[bibr15-00302228211016227] Prigerson H. G. Horowitz M. J. Jacobs S. C. Parkes C. M. Aslan M. Goodkin K., Raphael, B., Marwit, S. J., Wortman, C., Neimeyer, R. A., Bonanno, G., Block, S. D., Kissane, P., Boelen, P., Maercker, A., Litz, B. T., Johnson, J. G., First, M. B., & Maciejewski P. K. (2009). Prolonged grief disorder: Psychometric validation of criteria proposed for DSM-V and ICD-11. PLoS Medicine, 6(8), e1000121. 10.1371/journal.pmed.1000121 19652695 PMC2711304

[bibr16-00302228211016227] Simon N. M. Saxe G. N. Marmar C. R. (2020). Mental health disorders related to COVID-19-related deaths. Journal of the American Medical Association, 324(15), 1493–1494. 10.1001/jama.2020.19632 33044510 PMC11404536

[bibr17-00302228211016227] Stroebe M. Schut H. (2020). Bereavement in times of COVID-19: A review and theoretical framework. OMEGA-Journal of Death and Dying, 82(3), 500–522. 10.1177/0030222820966928 PMC7756059 33086903

[bibr1501-00302228211016227] Sun, Y., Bao, Y., & Lu, L. (2020). Addressing mental health care for the bereaved during the COVID-19 pandemic. *Psychiatry and Clinical Neurosciences, 74*(7), 406–407. doi:10.1111/pcn.13008 10.1111/pcn.13008 32303110

[bibr18-00302228211016227] Tang S. Xiang Z. (2021). Who suffered most after deaths due to COVID-19? Prevalence and correlates of prolonged grief disorder in COVID-19 related bereaved adults. Globalization and Health, 17(1), 19. 10.1186/s12992-021-00669-5 PMC7877329 33573673

[bibr19-00302228211016227] Tang S. Yu Y. Chen Q. Fan M. Eisma M. C. (2021). Correlates of mental health after COVID-19 bereavement in Mainland China. *Journal of Pain and Symptom Management*. Advance online publication. 10.1016/j.jpainsymman.2021.02.016 33662513

[bibr20-00302228211016227] Wallace C. L. Wladkowski S. P. Gibson A. White P. (2020). Grief during the COVID-19 pandemic: Considerations for palliative care providers. Journal of Pain and Symptom Management, 60(1), e70–e76. 10.1016/j.jpainsymman.2020.04.012 PMC7153515 32298748

